# The relationship between objective app engagement and medication adherence in asthma and COPD: a retrospective analysis

**DOI:** 10.1038/s41598-021-03827-2

**Published:** 2021-12-21

**Authors:** Leanne Kaye, Rahul Gondalia, Alesha Thompson, David A. Stempel, Meredith A. Barrett

**Affiliations:** 1 ResMed Science Center, San Diego, CA USA; 2 CDC Foundation, Atlanta, GA, USA; 3 Propeller Health, San Francisco, CA, USA

**Keywords:** Asthma, Chronic obstructive pulmonary disease, Human behaviour

## Abstract

Digital health tools can promote disease self-management, but the association of smartphone app engagement and medication adherence is unclear. We assessed the relationship between objective smartphone app engagement and controller medication use in adults with asthma and COPD. We retrospectively analyzed data from participants enrolled in a digital self-management platform for asthma and COPD. Eligible adults had a smartphone and a paired electronic medication monitor (EMM). Longitudinal, mixed-effects logistic regressions estimated the relationship between daily app engagement (app opens, session duration) and daily controller medication use. Data from 2309 participants (71% asthma; 29% COPD) was analyzed. Opening the app (vs. not opening the app) was associated with significantly greater odds (OR (95% CI)) of using controller medications in asthma (2.08 (1.98, 2.19)) and COPD (1.61 (1.49, 1.75). Longer session duration was also associated with greater odds of using controller medications in asthma and COPD, but the odds of use attenuated with longer session duration in COPD. This study presents a novel assessment of the relationship between objectively-measured smartphone app engagement and controller medication use in asthma and COPD. Such insights may help develop targeted digital health tools and interventions.

## Introduction

In the United States, it is estimated that 24.7 million children and adults have asthma^1^, and more than 16 million adults have COPD^2^. While distinct diseases, patients with asthma and COPD may experience similar symptoms like shortness of breath, chest discomfort and frequent cough^3^.

Inhaled controller medications help patients manage their asthma and COPD and may improve patient quality of life. Yet, adherence to these daily inhalers is frequently suboptimal, with estimates of controller adherence averaging 30–50% for patients with asthma^4^ and less than 50% for patients with COPD^5,6^. Medication non-adherence may lead to poor patient outcomes and be costly to patients and the healthcare system more broadly^7–9^.

Use of smartphone self-management applications (“apps”) have demonstrated value in helping people with asthma and COPD improve or maintain good medication adherence^10–12^ especially if behavior change techniques like feedback, education and self-monitoring are integrated and address core barriers to poor adherence^13–15^. A systematic review of digital interventions for asthma self-management found that digital programs integrating theory-based behavioral change approaches significantly improved medication adherence compared to those that did not^16^.

Despite this encouraging evidence, the impact of digital tools like smartphone apps can be limited by challenges not only in retention, but in engagement as well^17^. Further, self-reported engagement can be prone to bias and limited in what it can measure^18,19^. As such, it remains unclear what the relationship between app engagement and medication adherence is, especially in asthma and COPD. Thus, we aimed to explore the relationship between app engagement and medication adherence using objective data collected from a real-world sample of adults with asthma and COPD enrolled in a digital health platform.

## Methods

### Recruitment and eligibility

Data used in this retrospective analysis was collected from participants who enrolled in a digital self-management platform (Propeller Health, WI, USA) between January 2018 and March 2019. Participants who enrolled in the platform were recruited via social media campaigns (e.g., Facebook advertisements), and needed to own a smartphone and have a self-reported history of asthma and/or COPD to be eligible. All participants agreed to the platform’s Terms of Service.

Data was examined retrospectively using an aggregated dataset. To be included in the analysis, participants needed to be ≥ 18 years of age, reside in the United States, and have a controller inhaler compatible with an electronic medication monitor (EMM). Participants also needed to have at least 97 days of controller medication use data (the first seven days of participation was considered an onboarding period). The retrospective analysis plan was determined to be exempt and consent was waived by the Copernicus Independent Review Board (PRH1-18-132). A subset of data used in this retrospective analysis included data previously collected from an electronic survey to which patients provided consent (Protocol 20191728). All methods were carried out in accordance with relevant guidelines and regulations.

### Description of the digital health platform

**Figure 1:**
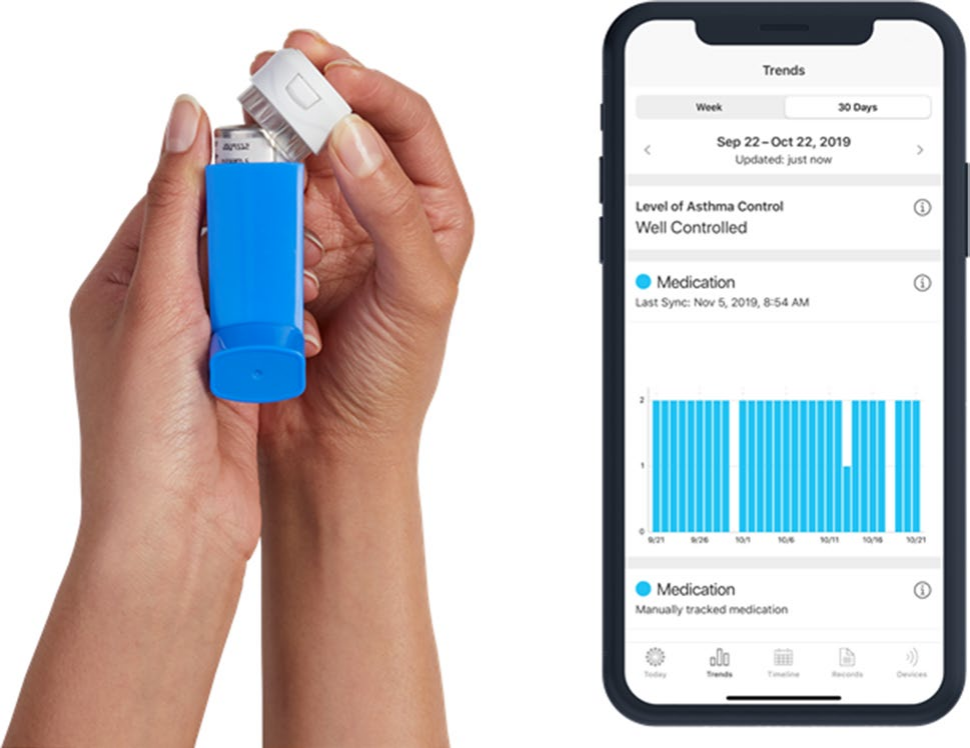
A small FDA-cleared electronic medication monitor (EMM) is attached to the user’s controller medication inhaler to capture the date and time of use. Data from the EMM is then transferred wirelessly via Bluetooth to a paired smartphone app providing feedback, insights and medication reminders.

Propeller Health is an FDA-cleared digital platform that includes an EMM, and a paired smartphone app targeted to the user’s self-reported condition (asthma or COPD) (Fig. 1).

Electronic medication monitors (EMMs) attach to a compatible inhaler medication to passively capture the date and time of use. Usage data is then wirelessly transferred via Bluetooth to a paired smartphone app. The EMM has a battery life of 12–18 months and does not require charging^11^. Compatible medications include inhaled corticosteroids (ICS), long-acting beta-agonists (LABA), long-acting muscarinic antagonists (LAMA), and combination therapies (ICS + LABA, LABA/LAMAs and triple therapy).

The paired smartphone app serves not only to collect data from the EMM, but also to engage the user through evidence-based asthma and/or COPD content, including relevant guideline content^20,21^, feedback on medication use and trends, and schedule-based medication reminders through the EMM and smartphone application. Patients with continued poor medication adherence may be presented with additional gamified features and challenges aimed at improving daily medication adherence. The app also prompts users to complete an in-app Asthma Control Test (ACT) or COPD Assessment Test (CAT) at baseline and monthly thereafter to better assess disease control and burden, respectively (Supplementary Fig. 1a,b).

### Outcome and measures

#### Smartphone app engagement

We examined app engagement, defined by daily app opens and app active session duration, as the independent variable. Daily app engagement was defined differently for each model estimate to explore the varying associations of duration on controller medication adherence: Model 1 (no app open vs. any app open regardless of session duration) and Model 2 (no app open vs. at least one app open with < 1, 1–< 5, 5–10 and 10+ minutes of total daily app session duration).

#### Controller medication use

Controller inhaler use was determined using data collected from the EMM, which recorded the date and time of each inhaler actuation. Adherence was calculated by dividing the number of EMM-recorded actuations by the prescribed number of actuations (reported by the participant during enrollment) × 100 per day.

For the primary analyses, the outcome of controller medication use was dichotomously defined as having taken at least 1 actuation vs. no actuation per day. Controller adherence was dichotomized because daily adherence was either 0% or 100% on the majority of participant-days (74%). For secondary analyses, controller medication use was defined as being 100% adherent vs. < 100% per day. Being 100% adherent was defined as having EMM-recorded controller actuations that were greater than or equal to the total number of prescribed actuations for that day.

#### Self-reported measures of control and burden in asthma and COPD

Symptom control was assessed with the Asthma Control Test (ACT), a self-administered, validated questionnaire for patients with asthma. The 5-item assessment asks respondents to rate their symptoms on a scale of 1–5. A score > 19 indicates good symptom control, 15–19 indicates not well-controlled symptoms, and < 15 indicates very poorly controlled symptoms^22^.

Disease burden for participants with COPD was assessed with the COPD Assessment Test (CAT). The 8-item self-administered questionnaire asks respondents to rate their symptoms on a scale of 1–5. Summative scores < 20 indicate lower disease burden, and scores ≥ 20 indicate higher disease burden^23^.

### Analyses

Longitudinal, mixed-effects logistic regressions were used to estimate the association between daily app engagement and daily controller medication adherence during days 8–97 of EMM use for asthma and COPD, adjusting for age, gender, smartphone type (iOS vs. Android), baseline disease status (defined as initial ACT or CAT score), rescue medication use, day on platform and US Census-derived neighborhood-level income and education. Analyses were also stratified by age and disease severity. Stratification by age was completed for participants 40 years and older to allow for comparisons between asthma and COPD. Odds ratios and 95% confidence intervals (CI) were presented, with alpha = 0.05 as the significance threshold.

#### Role of funding source

Representatives from the study sponsors (Propeller Health, Council of State and Territorial Epidemiologists) were involved in the study design, collection, analysis and interpretation of data, writing of the report, and in the decision to submit the paper for publication. The corresponding author had full access to all the data in the study and had final responsibility for the decision to submit for publication.

## Results

### Demographics

There were 2309 participants included in the analysis of whom 71% (N = 1629) had self-reported asthma, and 29% (N = 663) had self-reported COPD. Participants with asthma had a mean age (SD) of 39 (12) years and 80% were female. Participants with COPD had a mean (SD) age of 60 (9) years and 67% were female. At baseline, participants with asthma self-reported very poor control (mean ACT score: 13.2) and participants with COPD reported higher COPD burden (mean CAT score: 23.7) (Table 1).

**Table 1:** Participant characteristics*

	Asthma n = 1629	COPD n = 663
Age (mean (SD)); years	39.4 (12.6)	60.9 (8.3)
Female, n (%)	1302 (80)	443 (67)
Baseline CAT, mean (SD)	–	23.8 (7.5)
Baseline ACT, mean (SD)	13.3 (4.5)	–
Uncontrolled Asthma (ACT < 20), n (%)	1448 (90.1)	
Higher Burden COPD (CAT > 20), n (%)		449 (67.7)
Android, n (%)	860 (53)	437 (66)
Rescue use (mean (SD)), puffs/day	1.0 (1.8)	1.74 (2.5)
Daily medication adherence (mean (SD)), %	45 (32)	62 (32)
Percent of days with 100% adherence (mean (SD)), %	31 (33)	50 (38)
Percent of days with app opens (mean (SD)), %	16 (19)	28 (27)
App opens/day (mean (SD))	0.2 (0.4)	0.5 (0.8)
Daily app session duration (mean (SD)), min^a^	4.4 (6.5)	4.3 (5.0)

*90 days of participant data included e.g., mean rescue puffs/day was calculated over 90 days.

^a^Capped at 60 min/day.

### App opens, session duration and medication adherence

Over 90 days, unadjusted mean (SD) medication adherence was higher among participants with COPD than participants with asthma: 62 (32)% vs. 45 (32)%, respectively. On average, participants with asthma and COPD spent a similar amount of time in the app per day: 4.4 (6.5) min compared to 4.3 (5.0) min, respectively. Participants with COPD opened their app more often than participants with asthma (mean daily app opens (SD): 0.5 (0.8) vs. 0.2 (0.4)).

In participants with asthma (n = 1629), opening the app on a given day was associated with higher odds of using controller medication compared to participants who did not open the app; OR (95% CI) 2.08 (1.98, 2.19), p < 0.001. Participants who spent more time in the app were more likely to use their controller medication compared to those participants who spent no time in the app, and the odds of controller use was higher with greater app duration: < 1 min: OR (95% CI) 1.95 (1.82, 2.08) vs. > 10 min OR (95% CI) 2.70 (2.32, 3.14) (Table 2).

**Table 2:** Odds ratios between any app open (reference = no app use) and any daily controller medication use, 90 days (n (asthma) = 1629, n (COPD) = 663).

Model	Odds ratio (ref = no app open)	Odds ratio	Lower 95% CI	Upper 95% CI	*p*
**Asthma**					
1	Any app open	2.08	1.98	2.19	< 0.001
2	< 1 min duration	1.95	1.82	2.08	< 0.001
	1–< 5 min duration	2.14	1.97	2.32	< 0.001
	5–10 min duration	2.30	1.93	2.75	< 0.001
	10+ min duration	2.70	2.32	3.14	< 0.001
**COPD**					
1	Any app open	1.61	1.49	1.75	< 0.001
2	< 1 min duration	1.87	1.69	2.07	< 0.001
	1–< 5 min duration	1.38	1.23	1.54	< 0.001
	5–10 min duration	1.58	1.26	1.98	< 0.001
	10+ min duration	1.34	1.10	1.61	0.003

All generalized linear mixed effects logistic models adjusted for census-level income and education, age, gender, android (vs. IOs), ACT (for asthma) or CAT (for COPD) score, days since first controller EMM sync, and included a random intercept for participant to account for repeated measures.

In participants with COPD (n = 663), opening the app on a given day was also associated with higher odds of using controller medications compared to those participants who did not open the app (OR (95% CI) 1.61 (1.49, 1.75); p < 0.001). Participants who spent any amount of time in the app were more likely to use their controller medication compared to those who spent no time in the app; however, the magnitude of the odds of using controller medications per day attenuated with longer durations spent in the app: < 1 min OR (95% CI) 1.87 (1.69, 2.07) vs. > 10 min OR (95% CI) 1.34 (1.10, 1.61) (Table 2).

Similar patterns were observed in secondary analyses examining association with 100% controller adherence in participants with asthma and COPD (Supplementary Table 1).

Differences by age were observed for session durations of 5–10 min in both asthma and COPD, such that participants 40–60 years had higher odds of using controller medication than those > 60 years. Differences by disease severity were not observed for either asthma or COPD (Supplementary Tables 2, 3).

## Discussion

This study demonstrates that in a large, real-world sample of adults with asthma or COPD using a digital health platform, greater smartphone app engagement is associated with higher absolute medication use. Specifically, we observed a significant relationship between controller medication use and daily app opens as well as the length of time spent in the app. Differences in app usage by disease type were also observed such that participants with COPD opened the app more often, but had diminishing benefit from prolonged session durations (10+ minutes) compared to participants with asthma.

At present, research on objective measures of smartphone app behavior and its relation to desired outcomes in respiratory disease management is relatively limited. However, research in other chronic conditions have demonstrated similar positive relationships between objective app engagement and positive behavior change. For example, in a small randomized trial promoting physical activity, Edney^24^ found that participants with high levels of app engagement, defined as daily app login and feature interaction, completed significantly more minutes of physical activity than those with lower levels of app engagement. In another study examining app engagement across multiple mobile applications for mental health, frequency of app use as well as lifetime app use was positively associated with improvements in depression and anxiety^25^. While these studies support our initial findings, more evidence is needed in asthma and COPD.

In our study, we examined outcomes by any medication use (< 100% of prescribed doses) (Table 2) and by 100% medication use (Supplementary Table 1). We observed that the odds of meeting 100% medication use were attenuated compared to odds of meeting < 100% medication use in both asthma and COPD when opening the app. The same trend was observed for session duration. This finding is not surprising, as patients who achieve their behavioral goal may be more motivated, and have greater self-efficacy^26^, and as such may not have relied on the app features to achieve their goals.

We also observed that the likelihood of using controller medications increased with longer session duration among participants with asthma, but the same did not hold true for those with COPD even when controlling for age. While we did not assess the change in app usage over time, it is possible that frequency of app use, not necessarily longer app sessions durations, could help explain the relationship observed, especially given that participants with COPD had a higher number of mean app opens per day. The literature also suggests that knowledge can play an important role in promoting medication adherence^27^. As such, it’s possible that participants with COPD were better educated about their condition (possibly due to more clinical visits and consequently, education) and may have relied more heavily on other features of the digital platform not requiring them to open the app, e.g., the audio-visual reminders on the EMM or the app-based push notifications. Further investigation is needed to confirm these hypotheses.

Finally, we observed some age-related variability among older adults (40–60 vs. 60 years of age) for both asthma and COPD, but its interpretation is unfortunately limited by smaller sample sizes. Further, while we did not observe a moderating effect by disease severity, there is evidence suggesting that patients who do not meet their behavioral goals are less likely to engage in that target behavior^28^. As such, it is possible that level of adherence to medication, rather than disease severity, may have moderated engagement so that those participants who met their adherence goals were more likely to engage with the app, than those who did not meet their adherence goals.

### Limitations

This retrospective analysis is an important first step in better understanding objective measures of smartphone app engagement, but there are limitations. First, we did not have access to participant-level demographic information such as race or socioeconomic status, which may be important determinants of engagement and medication adherence. Second, while we measured two objective measures of engagement, other objective measures like app push notifications and reminders, daily and monthly active user ratios (DAU/ MAU)^29^, number of in-app clicks, opening of app content pages, etc. may also provide useful signals. Third, following examination of the data for the secondary analysis, we decided posteriori to assess medication use as a dichotomous outcome instead of a continuous variable due to the non-normal distribution. Fourth, the generalizability of the results may be limited given that the majority of participants were female (research suggests that women tend to be more engaged than men in digital platforms^30^), and that participants who enrolled in the platform were potentially more motivated to improve their health. Finally, data on behavior change determinants of medication adherence were not collected, limiting insight into the behavior change pathways leading to the outcomes observed in the analysis^31^. Further, we did not examine outcomes by dosing schedule, nor were participants asked to report if they had shared their data with their healthcare provider^32^ both of which could be important moderating factors.

## Conclusion

Using objective app usage data, this retrospective analysis demonstrates how use of a smartphone app for asthma and COPD management is associated with medication adherence. Future work should explore additional measures of smartphone app engagement, as well as measure and assess behavior change pathways that may impact medication use over time.

## Supplementary Information

Supplementary Figure.

Supplementary Tables.

## Data Availability

Individual participant data will not be available publicly or at request.

## Abbreviations

ACT: Asthma control test
CAT: COPD assessment test
COPD: Chronic obstructive pulmonary disease
CI: Confidence interval
EMM: Electronic medication monitor
SD: Standard deviation
OR: Odds ratio
