# Supplementary material for: The relationship between objective app engagement and medication adherence in asthma and COPD: a retrospective analysis
Source: Sci Rep. 2021 Dec 21;11:24343. doi: 10.1038/s41598-021-03827-2 (PMC8692590; doi:10.1038/s41598-021-03827-2)
Supplement: Supplementary file 1 — Supplementary Figure. [file 41598_2021_3827_MOESM1_ESM.docx]

**Supplementary Figure**

a)
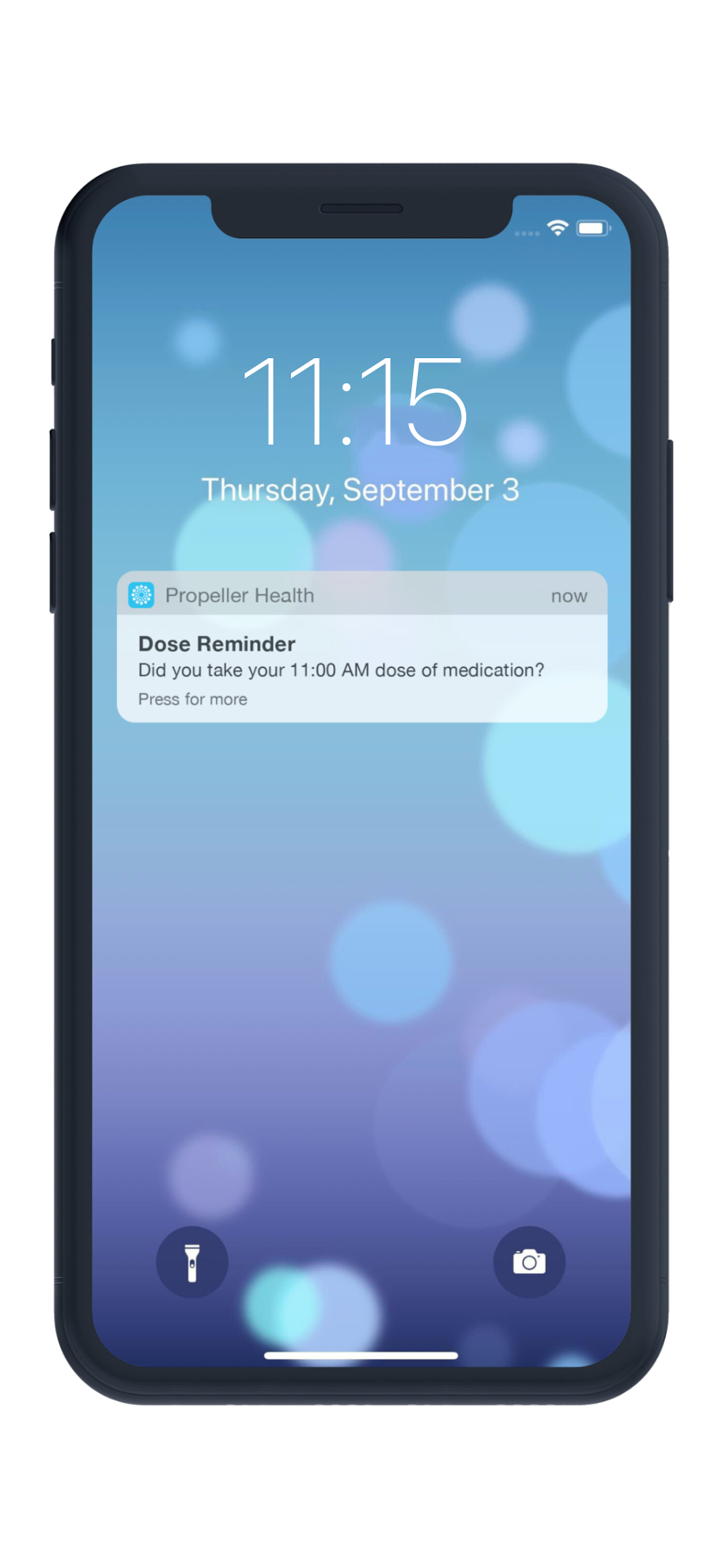
 b)
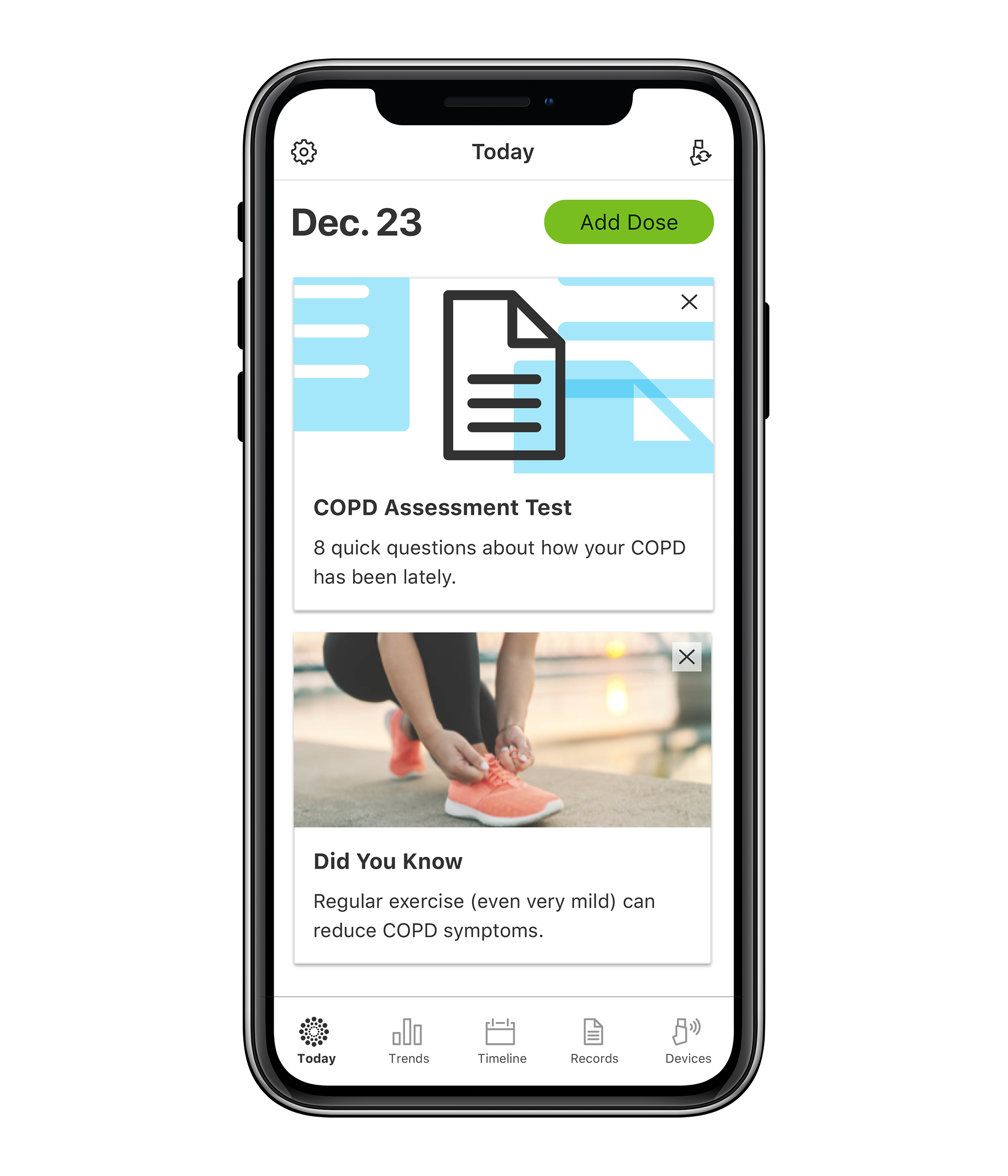


**Supplementary Figure 1a-b:** a) Example of a push notification to remind a participant to take their medication, and b) Example of the in-app questionnaire (in this case, the CAT for COPD participants) and delivery of evidence-based education tools.
