# Supplementary material for: The relationship between objective app engagement and medication adherence in asthma and COPD: a retrospective analysis
Source: Sci Rep. 2021 Dec 21;11:24343. doi: 10.1038/s41598-021-03827-2 (PMC8692590; doi:10.1038/s41598-021-03827-2)
Supplement: Supplementary file 2 — Supplementary Tables. [file 41598_2021_3827_MOESM2_ESM.docx]

**Supplementary Table 1:** Odds ratios between any app open (reference = no app use) and 100% adherence to daily controller medications, 90 days (n(asthma) = 1,629, n(COPD) = 663)

|  |  |  |  |  |  |  |
| --- | --- | --- | --- | --- | --- | --- |
|  | Model | Odds ratio (ref = no app open) | Odds ratio | Lower 95% CI | Upper 95% CI | *P* |
| Asthma | 1 | Any app open | 1.48 | 1.41 | 1.55 | < 0.001 |
|  | 2 | <1 min. duration | 1.42 | 1.34 | 1.51 | < 0.001 |
|  |  | 1-<5 min. duration | 1.49 | 1.38 | 1.60 | < 0.001 |
|  |  | 5-10 min. duration | 1.51 | 1.29 | 1.77 | < 0.001 |
|  |  | 10+ min. duration | 1.77 | 1.55 | 2.02 | < 0.001 |
| COPD | 1 | Any app open | 1.30 | 1.22 | 1.40 | < 0.001 |
|  | 2 | <1 min. duration | 1.45 | 1.33 | 1.59 | < 0.001 |
|  |  | 1-<5 min. duration | 1.23 | 1.12 | 1.36 | < 0.001 |
|  |  | 5-10 min. duration | 1.13 | 0.94 | 1.35 | 0.195 |
|  |  | 10+ min. duration | 1.04 | 0.88 | 1.22 | 0.653 |

All generalized linear mixed effects logistic models adjusted for census-level income and education, age, gender, android (vs. iOS), ACT (for asthma) or CAT (for COPD) score, days since first controller EMM sync, and included a random intercept for participant to account for repeated measures

**Supplementary Table 2:** Sensitivity analyses by age and disease severity* in asthma.

|  | **Model** | **Odds ratio**  **(ref = no app open)** | **Odds ratio** | **Lower 95% CI** | **Upper 95% CI** | **P** |
| --- | --- | --- | --- | --- | --- | --- |
| **40+ years of age** | 1 | Any app open | 1.91 | 1.78 | 2.05 | < 0.001 |
|  | 2 | <1 min. duration | 1.79 | 1.64 | 1.96 | < 0.001 |
|  |  | 1-5 min. duration | 1.98 | 1.78 | 2.21 | < 0.001 |
|  |  | 5-10 min. duration | 2.03 | 1.60 | 2.58 | < 0.001 |
|  |  | 10+ min. duration | 2.32 | 1.90 | 2.82 | < 0.001 |
| **40-60 years of age** | 1 | Any app open | 2.06 | 1.90 | 2.23 | < 0.001 |
|  | 2 | <1 min. duration | 1.89 | 1.72 | 2.09 | < 0.001 |
|  |  | 1-5 min. duration | 2.16 | 1.91 | 2.45 | < 0.001 |
|  |  | 5-10 min. duration | 2.78 | 2.09 | 3.70 | < 0.001 |
|  |  | 10+ min. duration | 2.42 | 1.93 | 3.04 | < 0.001 |
| **60+ years of age** | 1 | Any app open | 1.33 | 1.12 | 1.58 | < 0.001 |
|  | 2 | <1 min. duration | 1.34 | 1.08 | 1.65 | < 0.001 |
|  |  | 1-5 min. duration | 1.34 | 1.05 | 1.71 | < 0.001 |
|  |  | 5-10 min. duration | 0.79 | 0.51 | 1.22 | < 0.001 |
|  |  | 10+ min. duration | 1.86 | 1.25 | 2.77 | < 0.001 |
| **Asthma, all ages, well controlled (ACT > 19)** | 1 | Any app open | 1.75 | 1.49 | 2.07 | < 0.001 |
|  | 2 | <1 min. duration | 1.71 | 1.39 | 2.11 | < 0.001 |
|  |  | 1-5 min. duration | 1.59 | 1.24 | 2.05 | < 0.001 |
|  |  | 5-10 min. duration | 4.60 | 2.26 | 9.37 | < 0.001 |
|  |  | 10+ min. duration | 1.77 | 1.15 | 2.73 | 0.009 |
| **Asthma, all ages, not well controlled (ACT 15-19)** | 1 | Any app open | 2.18 | 1.94 | 2.44 | < 0.001 |
|  | 2 | <1 min. duration | 2.15 | 1.86 | 2.48 | < 0.001 |
|  |  | 1-5 min. duration | 2.14 | 1.78 | 2.57 | < 0.001 |
|  |  | 5-10 min. duration | 2.30 | 1.54 | 3.45 | < 0.001 |
|  |  | 10+ min. duration | 2.51 | 1.73 | 3.65 | < 0.001 |
| **Asthma, all ages very poorly controlled (ACT < 15)** | 1 | Any app open | 2.10 | 1.98 | 2.24 | < 0.001 |
|  | 2 | <1 min. duration | 1.92 | 1.77 | 2.08 | < 0.001 |
|  |  | 1-5 min. duration | 2.22 | 2.02 | 2.45 | < 0.001 |
|  |  | 5-10 min. duration | 2.14 | 1.74 | 2.63 | < 0.001 |
|  |  | 10+ min. duration | 2.93 | 2.45 | 3.50 | < 0.001 |

*Disease severity was determined by baseline ACT score: well controlled (ACT >19), not well controlled (ACT 15-19), and very poorly controlled (ACT <15)

**All generalized linear mixed effects logistic models adjusted for census-level income and education, age, gender, android (vs. IOs), ACT (for asthma) or CAT (for COPD) score, days since first controller EMM sync, and included a random intercept for participant to account for repeated measures

**Supplementary Table 3:** Sensitivity analyses by age and disease burden* in COPD

|  | **Model** | **Odds ratio**  **(ref = no app open)** | **Odds ratio** | **Lower 95% CI** | **Upper 95% CI** | ***P*** |
| --- | --- | --- | --- | --- | --- | --- |
| **COPD, 40-60 years of age** | 1 | Any app open | 1.81 | 1.61 | 2.04 | < 0.001 |
|  | 2 | <1 min. duration | 2.03 | 1.74 | 2.36 | < 0.001 |
|  |  | 1-5 min. duration | 1.52 | 1.27 | 1.81 | < 0.001 |
|  |  | 5-10 min. duration | 2.16 | 1.49 | 3.12 | < 0.001 |
|  |  | 10+ min. duration | 1.62 | 1.17 | 2.23 | < 0.001 |
| **COPD, 60+ years of age** | 1 | Any app open | 1.47 | 1.32 | 1.64 | < 0.001 |
|  | 2 | <1 min. duration | 1.75 | 1.53 | 2.00 | < 0.001 |
|  |  | 1-5 min. duration | 1.28 | 1.10 | 1.48 | < 0.001 |
|  |  | 5-10 min. duration | 1.27 | 0.95 | 1.69 | < 0.001 |
|  |  | 10+ min. duration | 1.18 | 0.93 | 1.50 | < 0.001 |
| **COPD, 40+ years of age, low burden (CAT<20)** | 1 | Any app open | 1.65 | 1.43 | 1.90 | < 0.001 |
|  | 2 | <1 min. duration | 1.94 | 1.62 | 2.33 | < 0.001 |
|  |  | 1-5 min. duration | 1.36 | 1.11 | 1.66 | < 0.001 |
|  |  | 5-10 min. duration | 1.59 | 1.06 | 2.39 | 0.026 |
|  |  | 10+ min. duration | 1.37 | 0.96 | 1.96 | 0.080 |
| **COPD, 40+ years of age, high burden (CAT ≥ 20)** | 1 | Any app open | 1.60 | 1.45 | 1.76 | < 0.001 |
|  | 2 | <1 min. duration | 1.84 | 1.62 | 2.08 | < 0.001 |
|  |  | 1-5 min. duration | 1.39 | 1.21 | 1.60 | < 0.001 |
|  |  | 5-10 min. duration | 1.57 | 1.20 | 2.07 | 0.001 |
|  |  | 10+ min. duration | 1.32 | 1.06 | 1.66 | 0.014 |

*Disease burden was determined by baseline CAT score: scores <20 indicate lower disease burden, and scores ≥20 indicate higher disease burden

**All generalized linear mixed effects logistic models adjusted for census-level income and education, age, gender, android (vs. IOs), ACT (for asthma) or CAT (for COPD) score, days since first controller EMM sync, and included a random intercept for participant to account for repeated measures
